# An evaluation of low volume high-intensity intermittent training (HIIT) for health risk reduction in overweight and obese men

**DOI:** 10.1186/s40608-017-0151-7

**Published:** 2017-04-19

**Authors:** Benjamin M. Kelly, Soteris Xenophontos, James A. King, Myra A. Nimmo

**Affiliations:** 1 Nuffield Health Research Group, Nuffield Health, 2 Ashley Avenue, Epsom, Surrey KT18 5AL UK; 2 University of Leicester, University Rd, Leicester, LE1 7RH UK; 3 Loughborough University, Epinal Way, Loughborough, LE11 3TU UK; 4 University of Birmingham, Birmingham, B15 2TT UK

**Keywords:** High-intensity intermittent training (HIIT), Exercise, Health, Obesity, Inflammation, Prevention

## Abstract

**ᅟ:**

Both sprint interval training (SIT) and high-intensity intermittent training (HIIT) have been described as time-efficient strategies for inducing favourable metabolic and cardiorespiratory adaptations in healthy and diseased participants.

**Background:**

To date, little attention has been given to profiling the potential health benefits of HIIT or modified HIIT training within overweight and obese cohorts with particular focus on inflammation. Within this pilot trial, we tested the hypothesis that 6 sessions of HIIT performed over 2 weeks with 1–2 days’ rest would improve aerobic capacity, glucose metabolism and inflammatory profile in an overweight and obese male cohort. Additionally, we profiled the potential health benefits of 4 HIIT sessions performed over the same period.

**Methods:**

18 overweight or obese males (BMI = 31.2 ± 3.6; V̇O_2_ = 30.3 ± 4.4 ml.kg.min^-1^) were studied before and 72 h after HIIT. Training sessions consisted of 10 x 1 min intervals at 90% HR_peak_ separated by 1 min recovery periods. Exercise was performed either 6 (group 1, *n* = 8) or 4 (group 2, *n* = 10) times over a 2 week period.

**Results:**

After training no changes were detected from baseline for body composition, aerobic capacity, glucose metabolism or inflammatory profile (*p* > 0.05) in either group.

**Conclusion:**

Both 6 and 4 sessions of HIIT performed over a 2-week period are ineffective in improving selected health markers within an overweight and obese cohort.

**Trial registration:**

This trial reports data from human participants and was retrospectively registered on 22/02/2017 with the ISRCTN registry, trial number ISRCTN90672085.

## Background

In overweight and obese individuals the core defect underlying the development of type 2 diabetes mellitus (T2DM) is skeletal muscle insulin resistance [1]. Mechanisms and primary contributing factors of insulin resistance are complex although evidence suggests that physical inactivity may be the principal initiating factor [2]. Inactivity leads to reduced energy expenditure, which when combined with increased energy intake promotes adipose tissue expansion and with it the development of obesity and a state of chronic inflammation [3]. Inflammation has been independently implicated in the development of insulin resistance and T2DM and is characterised by abnormal cytokine production, increased production of acute phase reactants as well as activation of a network of inflammatory signalling pathways [4, 5]. Regular exercise improves insulin sensitivity and is effective in preventing T2DM [6].

Traditionally, health-oriented physical activity guidelines have centred on moderate-intensity, continuous forms of exercise on most days of the week [7]. Although there are many perceived barriers to performing regular physical activity [8] one of the most commonly cited obstacles is lack of time [9] and when combined with recent evidence suggesting that some individuals prefer an intermittent exercise protocol in comparison to continuous exercise [10], it may be timely to consider novel forms of exercise that may be more readily adopted.

Previous work suggests that sprint interval training (SIT) in healthy populations provides a time-efficient strategy for inducing metabolic and cardiorespiratory adaptations comparable to those seen following traditional endurance based training [11–17]. Furthermore several authors have demonstrated that SIT and high-intensity interval training (HIIT), a moderately less intensive exercise modality, may have favourable effects on metabolic control after as few as 6 sessions in healthy [13, 14, 18–23], obese [24, 25], metabolic disease [26, 27], and heart failure participants [28]. Although positive effects have been previously demonstrated, it should be made clear that few trials cited here [11, 14] have utilised a short 2 week training period, instead longer durations have been employed which could have been responsible for health improvements.

Sprint interval training may be physically overreaching for sedentary and/or obese populations to complete efficiently. Recent modification of classic SIT exercise has led to more manageable HIIT training, which has shown to be metabolically effective. Little and colleagues [27] employed a manageable 2 week HIIT intervention within a T2DM cohort. Participants completed HIIT 3 x per week with exercise consisting of 10 x 1 min bicycle intervals at 90% of participants’ maximum heart rate (HR_max)_ whilst maintaining 80–100 RPM. Results demonstrated that with just a 75 min weekly training commitment key markers of glucose control were all significantly improved. Furthermore, the maximal workload achieved during a maximal cycling test increased by 10%. These data demonstrated that low-volume HIIT reduced hyperglycaemia and improved glucose tolerance whilst being well tolerated by a clinical population.

Exercise prescription is an important adjunct to clinical management in the prevention of cardio-metabolic disease [29]. While the traditional approach of prescribing moderate-intensity continuous exercise has been associated with improved health outcomes and a low incidence of adverse events [30], there is growing evidence for a dose–response relationship between exercise intensity and all-cause mortality, suggesting that higher-intensity exercise may afford greater benefit [31]. As such, we aimed to profile the aforementioned [27] HIIT intervention to assess if positive health improvements would be achieved in an overweight and obese cohort who may be at risk of developing cardio-metabolic disease. Specifically we aimed to look in detail at a broad spectrum of risk factors, including inflammatory markers that have, to date, received little attention in this specific context.

Furthermore, an early meta-analysis [32] indicated that with exercise intensities ~90% of maximum oxygen uptake (V̇O_2max_) with relatively short total exercise duration, 2 sessions per week could produce increases in V̇O_2max_ in individuals with low initial fitness levels. It is yet to be elucidated if this is true following HIIT within an overweight and obese cohort and if improvements can be seen in parameters other than V̇O_2max_. We therefore sought to evaluate a modified version of the above protocol with reduced weekly exercise volume.

## Methods

### Experimental Approach to Problem

**Fig. 1:**
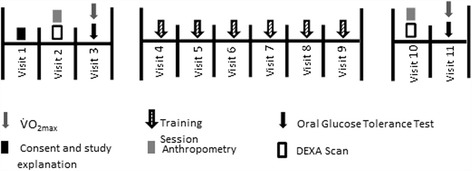
Schematic illustrating the experimental protocol. Visits 1, 2 and 3 took place before commencement of the HIIT. Visits 4-9 were spread over 2 weeks of training (i.e., 2 or 3 HIIT sessions per week with 24 - 72h between each session). Visits 10 took place 48 h after the last training session and visit 11 took place 72h after the last training session

Given the prominent exercise barrier of ‘time commitment’ we endeavoured to profile physiological changes associated with a reduced frequency variation of the aforementioned exercise protocol [27]. We hypothesised that previous findings will be replicated when conducted in an overweight and obese cohort, with glucose, insulin and inflammatory profiles expected to improve. Additionally, we expected to see improvements in V̇O_2peak_ following 4 exercise sessions in a 2-week period. For a experimental protocol please see Fig. 1.

### Participants

Full participant characteristics are provided in Table 1. Participants’ eligibility to take part in this study was determined during a pre-assessment session with a member of the research team. Herein, participants completed questionnaires assessing health status and physical activity habits. Blood pressure, BMI and fasting blood glucose (finger prick) were also assessed using a point-of-care analyser. All participants had a BMI ≥ 27 kg · m^-2^, reported taking part in any form of exercise less than 2 times per week, but were otherwise healthy. Participants were excluded if they were smokers, were diagnosed with impaired fasting glucose or diabetes, or had a BMI ≥ 40 kg.m^-2^.

**Table 1:** Body composition, blood pressure and peak oxygen uptake for groups 1 and 2 pre and post 2 weeks of HIIT

	Group 1 (6 HIIT sessions)			Group 2 (4 HIIT sessions)		
	Pre-Training	Post-Training	*p*	Pre-Training	Post-Training	*p*
	Mean (SD)	Mean (SD)	Sig. < 0.05	Mean (SD)	Mean (SD)	Sig. < 0.05
Body Mass (kg)	94.5 (13.9)	94.4 (14.3)	0.798	104.8 (17.6)	104.6 (17.0)	0.524
BMI (kg.m^2^)	29.7 (3.4)	29.7 (3.5)	0.716	32.3 (3.3)	32.3 (3.2)	0.557
Waist Circumference (cm)	100.4 (9.1)	99.1 (9.2)	0.164	109.6 (10.4)	108.6 (10.4)	0.316
Hip Circumference (cm)	107.6 (6.5)	106.9 (6.4)	0.235	113.9 (7.3)	112.7 (9.4)	0.499
Waist-to-hip ratio	0.9 (0.03)	0.9 (0.03)	0.709	1.0 (0.05)	1.0 (0.03)	0.879
Systolic BP (mmHg)	131.1 (15.8)	124.6 (10.5)	0.446	122.8 (8.2)	124.7 (5.2)	0.348
Diastolic BP (mmHg)	84.7 (10.9)	76.0 (9.7)	0.185	76.8 (7.1)	75.8 (5.4)	0.829
V̇O_2peak_ (l.min^-1^)	3.3 (0.7)	3.7 (0.8)	0.138	3.2 (0.5)	3.2 (0.4)	0.846
V̇O_2peak_ (ml.kg^-1^.min^-1^)	35.6 (5.4)	38.5 (5.4)	0.135	30.4 (4.2)	30.5 (3.9)	0.960

*BMI* body mass index; *BP* blood pressure

Group 1 (*N* = 8); Group 2 (*N* = 10)

### Procedures

#### Blood Pressure

Arterial blood pressure was measured using a digital automatic blood pressure monitor (Omron M7, Omron Healthcare UK Ltd, Milton Keynes, UK). Participants remained in a supine position for 10 min before the 1^st^ measurement. A cuff was placed around the upper dominant arm with participants’ arm rested on a firm surface during all measurements. Blood pressure was measured 3 times and the reported results are an average of the 3 readings.

#### Body Composition

Body mass (kg) was determined using a balanced beam scale (Seca, Hamburg, Germany) with height (cm) measured using an attached stadiometer (Seca, Hamburg, Germany) with participants wearing only shorts and no footwear. Participants had waist and hip circumferences measured with a measuring tape. Waist circumference was measured halfway between the iliac crest and the lowest rib. Hip circumference was measured at the widest part of the hips. These measurements were used to calculate the waist-hip ratio. Actual circumferences were determined from the average of two assessments at each site were both measurements being repeated in instances where measurements were more than 1 cm apart.

Total body composition was measured by dual-energy X-ray absorptiometry (DEXA) on a Lunar Prodigy (GE corporation, Connecticut, USA) which segmented the body into 3 compartments of fat mass, bone mineral content and fat-free soft tissue, the last 2 of which constitute fat-free mass and percent body fat. DEXA has been validated as a measure of body fat in overweight and normal weight individuals [33–35].

#### Oral Glucose Tolerance Test

Participants attended the laboratory having fasted for at least 12 h overnight. Plasma insulin and glucose were determined from venous blood samples collected from a 21 gauge cannula inserted into an antecubital vein. Blood samples were collected before, 30 min, 60 min, 90 min and 120 min after ingestion of 82.5 g dextrose monohydrate dissolved in 200 ml of water. This solution was immediately washed down with 100 ml of water. The cannula was kept patent via regular flushing with 0.9% (*w/v*) saline solution. The first 2 ml of blood extracted from the cannula via a syringe was discarded. Blood samples were collected into vacutainers (Becton Dickinson, Plymouth, UK) containing either 1.8 mg ethylenediaminetetraacetic acid (EDTA) per ml of blood (glucose and inflammatory hormones) or 17 IU lithium heparin per ml of blood (insulin). Blood samples were gently inverted 8 times and then placed on an SRT6 roller mixer (Bibby Scientific Ltd, Stone, UK) to ensure mixing.

Insulin and inflammatory blood samples were immediately centrifuged at 3500 g (10 min at 4 ^o^C) (Heraeus Labofuge 400 R, Langenselbold, Germany) and the plasma aliquoted into labelled eppendorf tubes and stored at -80^o^C until analysis. Whole blood glucose was analysed immediately using a glucose oxidase reaction via an automated analyser (YSI Stat 2300, Yellow Spring Instruments, Ohio, USA). The area under the curve (AUC) for plasma insulin and glucose were calculated from baseline (0 min) to 120 min after ingestion of the dextrose drink using the trapezoidal method. Tests were performed approximately 1 week prior to and exactly 72 h post exercise intervention.

#### Maximal Oxygen Uptake

V̇O_2peak_ was determined using a continuous incremental exercise test on an electromagnetically braked cycle ergometer (Lode Excalibur, Groningen, The Netherlands), performed to volitional exhaustion. Expired air was measured continuously using an online breath-by-breath gas analysis system (Cortex Metalyzer, CPX International Inc., Berlin, Germany). Participants warmed up for 5 min against a resistance of 50 W, after which the workload was increased linearly by 16 W per minute until the participant could no longer maintain 50 RPM. V̇O_2peak_ was identified as the highest value achieved over 15 breaths, taken from a rolling average. HR was measured throughout the test using a telemetric heart rate monitor, which was wirelessly paired with the breath-by-breath analysis system (Polar RS100, Polar Electro UK Ltd, Warwick, England).

### High Intensity Interval Training

The HIIT protocol utilised in this study was based on that devised by Little and colleagues [27]. Participants warmed up at a resistance of 50 W for 3 min and during the last 10 s participants counted down before the wattage was elevated to a pre-determined resistance set to elicit 90% HR_peak_. Resistance was manipulated manually throughout to ensure pre-determined heart rate values were achieved. During the 60 s high intensity interval, participants were asked to maintain a cadence of 80–100 RPM. After 60 s of high intensity cycling participants were instructed to cycle for the next 60 s at a cadence of 70–80 RPM against a resistance of 50 W (active recovery). This was repeated a further 9 times followed immediately by a 2 min cool down against a resistance of 50 W.

Group 1 (*N* = 8) completed 6 sessions of HIIT exercise over a 2-week period where as group 2 (*N* = 10) completed only 4 over the same period. Group 1 exercise sessions were carried out on Mondays, Wednesdays, and Fridays. Group 2 sessions were conducted on Mondays and Fridays.

### ELISAs and Biochemical Analysis

Adiponectin, MCP-1, IL-10, CRP and TNF-α were quantified using commercial sandwich enzyme linked immunosorbent assays (ELISAs) and TNF-α and IL-10 were measured via high sensitivity ELISAs (R & D systems, Minneapolis, MN, USA).

Plasma IL-6 and sIL-6R were analysed via ‘in-house’ ELISAs as detailed elsewhere [37, 38]. Materials and chemical reagents were obtained from Sigma-Aldrich Ltd (Poole, UK) unless otherwise specified. All incubation periods were at room temperature and during each incubation stage the plate was placed on a Stuart Mini Orbital Shaker (Bibby Scientifc Ltd, Stone, UK) at 60 revs.min^-1^ unless otherwise stated. Wash steps for ELISAs were carried out manually using an 8 way multi-channel pipette (BioHIIT eLINE, Helsinki, Finalnd). The absorbance of wells was read using a Varioskan Flash Mutimode Reader (Thermo Scientific, Vantaa, Finland). Protein concentration of samples was determined in relation to a 4-parameter logistic standard curve. All samples were analysed in duplicate and were repeated if the coefficient of variation (CV) between duplicates was more than 10%. The intra-assay CVs for the inflammatory proteins were as follows: adiponectin (3.5%), IL-10 (8.7%), TNF-α (7.8%), CRP (5.3%), IL-6 (4.8%), sIL6-R (3.5%), MCP-1 (6.4%).

### Insulin Sensitivity Index

Insulin sensitivity was estimated using the Matsuda index of insulin sensitivity [36] which is a validated measure which correlates highly (*r* = 0.73) with the rate of whole body glucose disposal during a euglycaemic-hyperinsulinaemic clamp.

### Statistical analysis

Statistical analysis was carried out using SPSS version 19 (SPSS Inc, an IBM company). All variables were checked for distribution with a Shapiro-Wilk test confirming normal distribution throughout. Statistical significance was assumed at *p* < 0.05. The primary outcome measure was the change in fasting glucose from pre- to post-intervention, with a clinically relevant difference between the interventions of 15%. Based on data on repeated measures of the oral glucose tolerance test (OGTT) test protocol, it is calculated that with a power of 80% and alpha set at 0.05, 8 participants are required per group to detect the minimal clinically relevant difference between the two interventions.

Pre to post-training differences in basal plasma glucose, insulin, glucose and insulin AUC, anthropometry and V̇O_2peak_ data were assessed using paired sample *t*-tests. Additionally, group differences were assessed via comparison of delta change using independent *t*-test. This approach was favoured over ANOVA due to the small sample size and because each group was to be evaluated for independent efficacy and not as a comparison between groups.

## Results

### Body composition, blood pressure and peak oxygen uptake

**Fig. 2:**
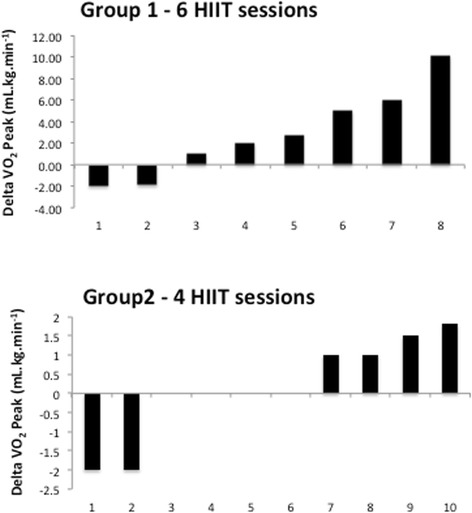
Participants’ individual V̇O_2peak_ responses to HIIT in group 1 (*top panel*) and group 2 (*bottom panel*)

There were no differences in characteristics between groups at baseline (Table 1). Following 6 sessions of HIIT over 2 weeks (Group 1), there were no changes in body mass, waist and hip circumferences or BMI (*p* > 0.05). Additionally no significant changes in V̇O_2peak_ were observed following HIIT in group 1, in absolute or relative terms. Figure 2 details individual changes in V̇O_2peak_ from baseline. These findings were replicated in group 2 who completed 4 sessions of HIIT over 2 weeks (Table 1).

**Fig. 3:**
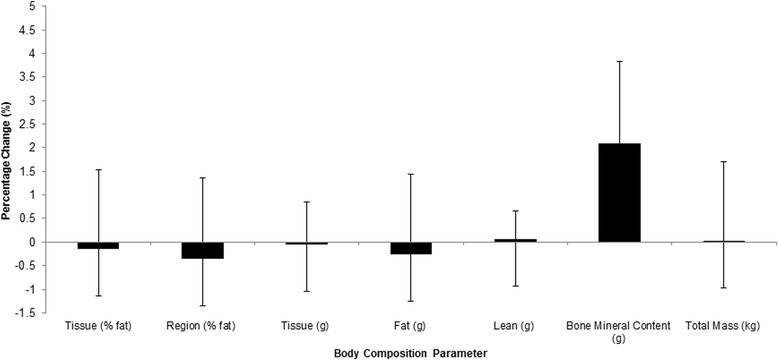
Group 1 percentage change in body composition parameters pre and post 2 weeks of HIIT

**Fig. 4:**
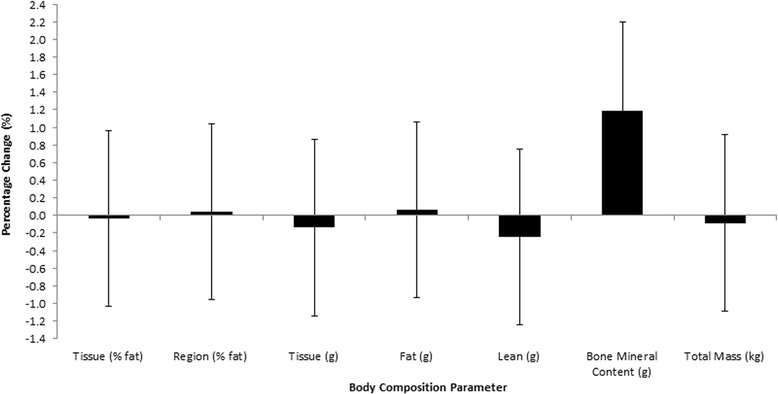
Group 2 percentage change in body composition parameters pre and post 2 weeks of HIIT

On assessment of DEXA data no changes in tissue and regional fat (%), total tissue (g), total lean tissue (g), total fat tissue (g) or bone mineral content (BMC) (g) were observed within or between groups (*p* > 0.05) (Figs. 3 and 4).

### Inflammatory proteins in the circulation at rest

After training there were no changes in plasma adiponectin, IL-10, TNF-α, IL-6, sIL-6R, CRP, or MCP-1 (*p* > 0.05) for either group (Table 2).

**Table 2:** Intra-assay coefficients of variance (CV) between duplicate samples for inflammatory protein analysis in plasma

	Group 1				Group 2			
	Pre-Training	Post-Training		*p*	Pre-Training	Post-Training		*p*
	Range (Median)	Range (Median)	Mean ∆Change	Sig. < 0.05	Range (Median)	Range (Median)	Mean ∆Change	Sig. < 0.05
Adiponectin (μg.ml^-^1)	1.1–14.6 (2.3)	0.8–10.9 (1.7)	−0.3	0.123	0.6–8.3 (2.2)	0.5–7.6 (1.9)	−2.0	0.158
IL-10 (pg.ml^-1^)	0.5–0.7 (0.5)	0.5–0.7 (0.5)	−0.03	0.304	0.4–0.8 (0.5)	0.5–1.0 (0.5)	−0.05	0.337
TNF-α (pg.ml^-1^)	1.4–2.6 (2.3)	1.1–3.2 (2.2)	−0.02	0.903	1.7–4.9 (2.5)	1.7–4.4 (2.5)	−5.4	0.704
CRP (μg.ml^-1^)	0.1–5.7 (1.5)	0.1–3.8 (1.5)	−0.3	0.358	0.2–4.0 (1.1)	0.1–3.6 (0.6)	−0.4	0.067
IL-6 (pg.ml^-1^)	1.6–112.4 (3.5)	1.3–87.0 (3.7)	−9.0	0.111	1.0–11.1 (2.8)	1.2–8.4 (2.6)	−0.5	0.182
sIL-6R (ng.ml^-1^)	21.3–36.3 (24.8)	19.8–32.3 (27.9)	1.2	0.518	21.7–51.0 (25.1)	23.6–65.3 (30.7)	4.2	0.246
MCP-1 (pg.ml^-1^)	51.4–411.1 (157.3)	55.8–181.0 (148.2)	−38.7	0.255	107.4–246.3 (143.5)	65.5−255.6 (141.3)	0.5	0.976

Inflammatory proteins in plasma for groups 1 and 2, pre and post 2 weeks of HIIT

Group 1 (*N* = 8); Group 2 (*N* = 10)

### Insulin sensitivity

**Fig. 5:**
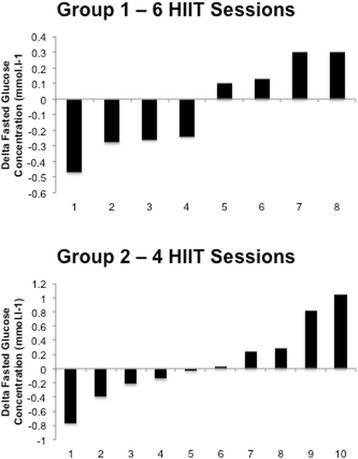
Participants’ individual basal fasted glucose responses to HIIT in group 1 (*top panel*) and group 2 (*bottom panel*)

**Fig. 6:**
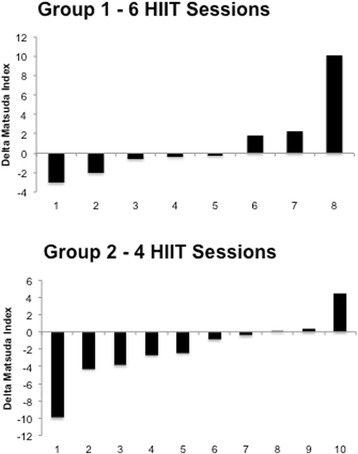
Participants’ individual insulin resistance (Matsuda Index) responses to HIIT in group 1 (*top panel*) and group 2 (*bottom panel*)

**Fig. 7:**
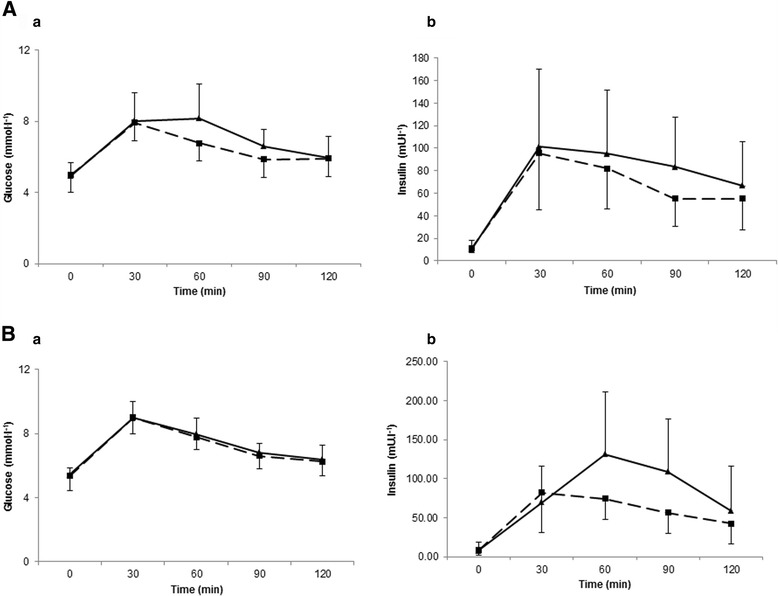
**a** Group 1 plasma glucose (*a*) and insulin (*b*) response to a 75 g OGTT pre and post 2 weeks of HIIT. (*dashed line*) represents pre-training. (*solid line*) represents post training. **b** Group 2 plasma glucose (*a*) and insulin (*b*) response to a 75 g OGTT pre and post 2 weeks of HIIT. (*dashed line*) represents pre-training. (*solid line*) represents post training

There were no significant changes in fasting glucose (Fig. 5), insulin or the insulin sensitivity index (Fig. 6) nor were there any differences found for the area under the curve in response to a 75 g OGTT in any group. The glucose and insulin responses to the 2 h OGTT before and after training is shown in Fig. 7a and b. Individual responses in insulin resistance are shown in Fig. 5.

## Discussion

The primary aim of this study was to profile the effectiveness of a previously described HIIT protocol [27], for improving aerobic capacity, glucose control and inflammatory profile in an overweight and obese cohort. Secondary to this we endeavoured to assess how the response differed when the protocol volume was reduced. This study has demonstrated that 3 sessions of submaximal high-intensity exercise per week is not sufficient for improving any of the aforementioned parameters in our overweight and obese cohort; this is also true following 2 sessions of HIIT per week over a 2 week period.

### Inflammation

Exercise of varying intensities may improve wellbeing and combat some of the basal increase in inflammation often associated with obesity, T2DM and cardiovascular disease [39–43]. The present investigation failed to identify any measurable changes in circulatory inflammatory proteins at rest following 2 weeks of HIIT in group 1 or group 2.

Data detailing the effects of SIT or HIIT on inflammatory status in an overweight or obese cohort is minimal. In a study that used a running mouse model, high-intensity training was associated with reduced pro-inflammatory and increased anti-inflammatory cytokine expression [44], implying that high-intensity exercise training might be more beneficial than moderate-intensity training in reducing the risk of chronic cardiovascular and metabolic diseases. This notion is supported by another study that demonstrated that a combination of high-intensity aerobic exercise, plus resistance exercise training, in addition to daily physical activity, is required to achieve a significant anti-inflammatory effect in T2DM patients [40].

Contrary to the present findings, Leggate and colleagues [41] demonstrated significant reductions in circulating sIL-6R, IL-6/sIL-6R complex, adiponectin and MCP-1 of approximately 10%, 13%, 11% and 12%, respectively, within an obese cohort. Given that the volume of HIIT used by Leggate was 4 times the volume of that in the current study it may be suggested that in order to reduce inflammatory profile, a minimal volume of HIIT must be attained.

### Glucose Metabolism

Inflammation has been independently implicated in the development of insulin resistance and T2DM [6] and is characterised by abnormal cytokine production, increased production of acute phase reactants as well as activation of a network of inflammatory signalling pathways [7]. Insulin stimulates tyrosine phosphorylation of insulin receptor signalling (IRS) proteins, which is a crucial event in mediating insulin action and is the primary signalling defect of systemic insulin resistance. Inflammatory mediators promote insulin resistance through inhibitory serine phosphorylation of IRS-1. IRS-1 serine phosphorylation disrupts insulin-receptor signalling through several distinct mechanisms, ultimately blocking insulin action [45].

Various studies have investigated the effects of 2 weeks of SIT or HIIT training on glucose metabolism [18, 22, 24, 27, 41, 46]. To the best of the author’s knowledge this study is the first to investigate HIIT together with reduced frequency HIIT training within an overweight and obese cohort.

In the present investigation there were no measurable changes in fasting glucose or insulin following 2 weeks of HIIT or reduced frequency HIIT. Our findings are in agreement with previous work after 2 weeks of SIT training [18, 22, 24]. Whyte and colleagues [24] failed to demonstrate changes in fasting glucose concentrations following 2 weeks (6 sessions) of SIT within an obese cohort however did show a significant 25% reduction in fasting plasma insulin concentrations. Similar adaptations were demonstrated by Hood and colleagues [46] following the same training intervention albeit in a sedentary population, defined as not having participated in a regular exercise programme for at least 1 year before the study. Leggate [41] showed no change in fasting glucose, insulin, insulin sensitivity index or AUC response to a 2 h OGTT after 2 weeks of HIIT.

We failed to demonstrate any change in area under the 2 h OGTT curve following HIIT or reduced HIIT. Contrary to our findings, 2 weeks of SIT [18, 24] and HIIT [27] have been shown to be effective in reducing area under the curve. Notably, Little et al [27] utilised a protocol identical to that adopted in the current study within a T2DM cohort. After 6 sessions of exercise, area under the 24 h blood glucose curve was reduced from 11,066 ± 1703 to 9572 ± 995 mmol^.^l^-1.^day^-1^. Whyte and colleagues [24] demonstrated similar findings, describing a significant reduction of 15% in 2 h insulin AUC following 2 weeks of SIT in an obese cohort.

With reference to insulin sensitivity indices, the current study is one of few to profile changes following 2 weeks of HIIT. No changes in insulin sensitivity (as measured via Matsuda index) were measured in either of the 2 experimental groups. These data contrast that of previous work which demonstrated significant improvements in insulin sensitivity following 2 weeks of SIT training [18, 22, 24]. Notably Hood and colleagues [46] indicated that after 2 weeks of HIIT, insulin sensitivity as measured by HOMA, increased significantly by 35% in a group of sedentary adults.

### Peak aerobic capacity

In the present study, groups 1 and 2 demonstrated no change in V̇O_2peak_ following training. These results are consistent with previous findings [12, 14, 19]. Contrary to our findings, other previous reports have revealed significant improvements in V̇O_2max_ [24, 41, 47, 48] following 2 weeks of training. Talanian and colleagues [48] demonstrated that following 6 HIIT sessions V̇O_2peak_ was increased in healthy women. Later Whyte [24] demonstrated significant improvements in V̇O_2max_ following 2 weeks of SIT within an obese population. The authors attributed significant improvements to a relatively low level of baseline fitness within their cohort. This is unlikely given that baseline aerobic capacity neither positively nor negatively associates with gains in exercise training induced maximal aerobic power [49, 50].

The work of Billat and colleagues clearly elucidates that improvements in V̇O_2max_ correlate highly with total time spent exercising at V̇O_2max_ [51]. Data detailing time spent at V̇O_2max_ during sub-maximal HIIT interventions is sparse making it difficult to relate training outcomes to this training parameter. Data from 30 s Wingate sprints demonstrates that trained individuals only spend between 18 and 22 s working at ≥ 90% V̇O_2max_ [52]. Typically this intensity was not achieved in the present study until repetition number 7, despite only a 7% drop in oxygen consumption by the end of 1 min recovery periods. Of note, once achieving peak intensity at repetition 7, participants failed to increase this in subsequent intervals. It is this short accumulative time spent at V̇O_2max,_ which makes it possibly not surprising that previous SIT and particularly submaximal HIIT studies such as ours, have failed to observe measurable changes in maximal aerobic power after 2 weeks.

Clearly intensity of exercise is a critical consideration when viewing responses to a training study. As per the work of Billat and colleagues the greater the accumulative time spent close to V̇O_2max_ the greater the benefits that are likely to be achieved in aerobic capacity. Previous work in similar populations utilising the same intensity have demonstrated significant improvements in V̇O_2max_ ranging from 8% to 35% [41, 53–56]. Non-surprisingly within these studies there is a clear trend between level of improvement and total training volume. Data is inconclusive as to whether training at intensities above those utilised in the current study lead to better outcomes in aerobic capacity. A number of studies [16, 24, 57–60] in similar populations to those in this work have demonstrated improvements comparable to those at lower intensities [41, 53–56]. It may be interesting to speculate therefore, that a threshold for potential adaptation is reached at approximately 80% V̇O_2max_, with further improvements governed by training volume. Laursen and colleagues [61] support this view, suggesting that a greater volume of intense exercise is required in order to effectively improve V̇O_2max_. What is clear from the present study is that the protocol utilised did not meet the hypothetical duration or intensity pre-requisites required to improve aerobic capacity.

### Body Composition

The current study is the first to incorporate both standard anthropometric measures and DEXA analysis before and after 2 week HIIT intervention of these volumes. Results indicated that no changes were detected for either group 1 or 2 in total body mass, waist or hip circumferences or waist:hip ratio. Furthermore, no changes were detected in tissue fat (%), regional fat (%), fat mass (g), lean mass (g) or bone mineral content (BMC) (g).

Waist circumference is an independent predictor of ectopic fat deposition and is one of the key screening variables used to identify those with metabolic syndrome [62]. To the best of our knowledge there have only been 2, 2 week SIT or HIIT interventions that note waist circumference changes [24, 41]. Both Whyte and Leggate describe reductions in waist circumferences of 2.4 cm and 1.4 cm respectively. These reductions in waist circumferences seem improbable after only 2 weeks especially without dietary restrictions; with average exercise energy expenditures of 735 kJ for Wingate sprints [63], 2788 kJ for a typical 60 min exercise session utilised by Leggate and colleagues and 1151 kJ for a typical session used within the current study [64]. It may be that variability within the measurement accuracy of waist circumference played a role in divergent data between pre and post intervention.

Our data are in agreement with previous work utilising longer HIIT periods lasting ~ 10 weeks [57, 58, 65]. Previous groups showed no changes in any anthropometric measurements following HIIT and equally saw no change in control groups performing continuous moderate-intensity exercise. Data from studies ranging between 3 and 6 months however show significant changes in BMI, body mass, body fat (%) and waist or hip circumferences [21, 53, 59, 66]. The equivalency of anthropometric changes in those studies may be attributable to high accumulative exercise energy expenditure. Notwithstanding it may be hypothesised that given an average energy expenditure of 1151 kJ per HIIT session for group 1 in the present study, and assuming that 36,000 kJ equates to 1 kg of fat, an individual may stand to ‘burn’ ~ 2.3 kg of fat over a 6 month period with group 2 likely to achieve ~ 50% of this. With this in mind, data then becomes comparable to that of previous longer-term HIIT interventions. These data would suggest that HIIT interventions longer than 3 months in duration are required in order to see beneficial changes in body composition, assuming no change in dietary intake.

## Conclusion

Existing data suggests that SIT and HIIT training can be effective in improving insulin sensitivity, body composition, V̇O_2max_ [67], and inflammation [66]. The current study demonstrates that not all short-term HIIT protocols are effective in providing significant health benefits. It remains unknown if a longer duration training period, utilising identical exercise protocols would be sufficient in improving cardio-metabolic health profile in the same cohort and therefore warrants further investigation.

Given the ever increasing diversity of exercise prescription, and indeed the urgency for low cost and scalable preventative health interventions, it is now vital that clinical practice optimises regimens for independent health outcomes and equally ensures that exercise design is specific to any given participant cohort.

We demonstrate that a protocol utilising 10 X 1 min intervals at 90% HR_peak_ with 1 min recovery periods is not sufficient in improving health markers within an overweight and obese group over this time course. This should be an active consideration for practitioners considering similar preventative interventions in this group. Further work building upon this trial should aim to evaluate participant groups whom demonstrate less favourable baseline metabolic characteristics. The present cohort demonstrated glucose, insulin and inflammatory values within healthy range which may therefore be a plausible reason for lack of findings in this trial.

## Abbreviations

°C: Degrees centigrade
AUC: Area under the curve
BMI: Body mass index
CM: Centimetre
CRP: C-reactive protein
CV: Coefficient of variation
DEXA: Dual-energy X-ray absorptiometry
EDTA: Ethylenediaminetetraacetic acid
GA: Gauge
GLUT: Glucose transporter
HIIT: High intensity intermittent training
HOMA: Homeostatic model assessment
HR: Heart rate
IL: Interleukin
KG: Kilogram
kJ: Kilojoule
MCP: Monocyte chemo-attractant protein
Min: Minute
ML: Millilitre
mRNA: Messenger ribose nucleic acid
OGTT: Oral glucose tolerance test
RPM: Revolutions per minute
S: Seconds
SIT: Sprint interval training
SPSS: Statistical Package for Social Sciences
T2DM: Type 2 diabetes mellitus
TNF: Tumour necrosis factor
VO2: Maximal volume of oxygen
W: Watts
